# Primer for Mainstreaming Mind-Body Techniques for Extreme Climates-Insights and Future Directions

**DOI:** 10.3390/medicines7030012

**Published:** 2020-03-06

**Authors:** Akshay Anand, Gurkeerat Kaur, Sridhar Bammidi, Deepali Mathur, Priya Battu, Kanupriya Sharma, Rahul Tyagi, Viraaj Pannu, Disha Bhanushali, Nitin Limaye

**Affiliations:** 1 Neuroscience Research Lab, Department of Neurology, Postgraduate Institute of Medical Education and Research, Chandigarh 160012, India; kaurgurkeerat6@gmail.com (G.K.); sri.bammidi@gmail.com (S.B.); priyabattu78@gmail.com (P.B.); sharmakanu993@gmail.com (K.S.); rahul15tyagi@gmail.com (R.T.); 2 School of Biotechnology, KIIT University, Bhubaneswar 751024, India; matdeepali@gmail.com; 3 Government Medical College and Hospital, Chandigarh 160030, India; viraajpannu@gmail.com; 4 Sri Sri institute of Advanced Research, Ved Vignan Maha Vidya Peeth, Bangaluru 560082, India; dhbhanushali@gmail.com

**Keywords:** acclimatization, acute mountain sickness, high altitude medicine, hypoxia-inducible factor, vascular endothelial growth factor, mind-body techniques (MBTs)

## Abstract

**Background**: The deprivation of oxygen reaching the tissues (also termed as hypoxia) affects the normal functioning of the body. This results in development of many diseases like ischemia, glaucoma, MCI (Mild Cognitive Impairment), pulmonary and cerebral edema, stress and depression. There are no effective drugs that can treat such diseases. Despite such failure, alternative interventions such as mind-body techniques (MBTs) have not been adequately investigated. **Methods**: The first part of this review has been focused on philosophical aspects of various MBTs besides evolving an ayurgenomic perspective. The potential of MBTs as a preventive non-pharmacological intervention in the treatment of various general and hypoxic pathologies has been further described in this section. In the second part, molecular, physiological, and neuroprotective roles of MBTs in normal and hypoxic/ischemic conditions has been discussed. **Results**: In this respect, the importance of and in vivo studies has also been discussed. **Conclusions**: Although several studies have investigated the role of protective strategies in coping with the hypoxic environment, the efficacy of MBTs at the molecular level has been ignored.

## 1. Introduction

Soldiers deployed at high altitude experience hardships due to adverse environmental circumstances. These harsh environmental conditions include hypoxia, cold temperature, inadequate food production, and distribution and production of high level of free radicals and oxidants [1]. To cope up with such environmental stress, the body acquires new biological survival adaptations characterized as acclimatization. If soldiers are kept posted there for longer durations, their biological system may be affected by physiological and/or possible epigenetic changes [1]. High altitude acclimatization studies describe the phenomena of regulated respiration and circulation. Whether anthropometric, physiological, biochemical, neurocognitive, and molecular changes occur in biological system, either by environmental stress or MBTs, has not been adequately studied.

Earlier studies have reported that constant dwelling at high-altitude results in the reduction of skin-fold thickness and limb circumference with little effect on the bodyweight [2]. Diastolic blood pressure, heart rate, and high-density lipoprotein (HDL) cholesterol levels have been found to be significantly increased when individuals are exposed to higher altitudes [3]. The deterioration in the quality of military operations, i.e., in the efficiency of performance and judgment ability of the military personnel, can impact their alertness, memory, reasoning, and perceptions [4]. This requires prompt or short duration intervention.

At high altitudes, tourists and newly posted soldiers usually suffer from hypoxia, which is a result of insufficient oxygen supply reaching the tissues. As mentioned, hypoxia may cause diseases like Ischemia [5], Glaucoma [6], MCI (Mild Cognitive Impairment) [7], pulmonary edema [8], stress and depression [9]. These diseases occur through a broad spectrum of changes occurring at the cellular and molecular level. Many of these mechanisms are related to decreased oxygen delivery which can cause mitochondrial dysfunction. This leads to the initiation of various cellular cascades such as an increase in reactive oxygen species as well as an increase in inflammatory mediators such as TNF-α, IL-1, and IL-6 through induction of NF-κB. Some other disease states such as Age-related macular degeneration may occur due to an upregulation of VEGF leading to an increase in angiogenesis. Still others such as Schizophrenia and Alzheimer’s disease may be influenced by telomerase activity, BDNF levels, hippocampal neurogenesis, and neuronal plasticity. The cost of treatment of these diseases including hospitalization and prescribed drugs is usually very high and, in most cases, the situation is rescued only by taking the affected person to lower heights. A survey conducted by World Health Organization in 2014 indicates that developed countries like United States spends 17.1 and Canada spends 10.4 of its total GDP (Gross domestic product) as compared to developing countries like India and Sri Lanka spending 4.7 and 3.5 respectively of its total GDP on healthcare [10]. It has been seen that acclimatization at higher altitudes involves gradual introduction of physical exercise. Physical exercise leads to increase in the blood corpuscular levels and facilitates adaptation to climate and pressure changes [11]. The role of physical exercise has been shown in adapting to hypobaric conditions [12]. Because physical exercise is not feasible means for all age and body types, alternative methods are required.

## 2. Results and Discussion

### 2.1. Philosophical Aspect of MBTs

Meditation, Yogic and other MBTs are fast drawing attention as preventive and affordable non-pharmacological interventions. Historically, “yoga” is of Sanskrit origin which means to join or to unite. It is a combination of physical, mental and spiritual practices that aim to achieve comprehensive wellbeing of an individual [13]. This includes *ashtanga yoga* (8 principles/limbs of *yoga*). It comprises of 8 limbs, where, first and the second limb of yoga are *yama;* refinement of the mind, body, and behavior of an individual and *niyama*; observances. Third and fourth limb of yoga includes *asana;* physical postures and *pranayama;* regulation of *pranic* energy (generating ‘vital’ energy by breathing) which helps in the enhancement of the immune system of an individual. Fifth and sixth limb of yoga are *pratyahara*; abstinence from the unnecessary distractions and *dharana;* concentration. Lastly, the seventh and eighth parts of yoga are *Dhyana;* inner enlightenment/meditation and *Samadhi;* self-realization, the ultimate goal of yoga [14]. Other than yoga, many ancient health practices are adapted to new requirements and one such innovation practice is *Sudarshan Kriya Yoga* (SKY) which involves an advanced form of rhythmic and controlled breathing processes with slow, medium and fast cycles. It aims for self-actualization through the process of purifying actions [15]. *Ujjayi* (victorious breath), and *bhastrika* (bellows breath) constitute *pranayamas* or the breathing techniques associated with SKY. It has been found to be effective in healing anxiety, depression and all types of stress and associated illnesses [16]. Here it is pertinent to introduce the *Ayurvedic* principles which are referred to in the ancient medical system such as *Prakriti/Tridosha* analysis (analysis of psychometric traits of an individual) which was recently validated in studies involving high altitude populations [17]. According to ayurvedic philosophy, *prakriti* (nature) which can be best described by his/her basic and the lifestyle regime of an individual can lead to the prediction or outcome of a disease [18]. *Tridosha* also refers to fundamental energies comprising of three different distinguishable physiological identities working simultaneously; *vata* (based on the principle of kinetic energy), *pitta* (principle of transformation energy) and *kapha* (principle of stabilizing energy) that are universal across the human body and mind and key to health outcomes. An individual does not inherit these three entities, but these are said to be the result of environmental influence during the prenatal development. Thus, every individual constitutes of a unique fraction of these entities, which determines the ‘*prakriti*’ of a person. These bio-energy centers work collectively by responding to the external environment for maintaining homeostasis and also govern one’s circadian rhythms. Change of pattern in the proportion and any impairment or disarrangement in the balance of these bio-energy centers lead to pathology or a diseased condition. Furthermore, the discovery of Single Nucleotide Polymorphism (SNPs) helps in differentiating all the three bio-energy centers and has been recently shown to be unique to each one of them. The genetic basis of *Prakrti* opens up a new dimension for personalized medicine [19].

Similarly, the three primary *gunas* (qualities) known as *tamas* (darkness), *rajas* (passion) and *sattva* (light /purity) emerge from this ethereal *prakriti,* creating the essentials of all nature-energy, matter and consciousness. Basically, *triguna* is tendency of one’s behavior expression. Prediction of general and contextual behavior on the basis of *gunas* is yet to be validated but hypothesized to be associated with hypoxia association with acclimatization [20]. Furthermore, scientific analysis has proved the effect of positive correlation of *Sattva* and the negative correlation of *rajas* and *tamas* with health [21].

*Prakriti* & g*una*-based analysis of the mind-body techniques (MBTs) performing subjects could be a useful tool to understand its physiological, clinical and molecular relationship. The detailed description of the phenotypic principles of health and disease state has been provided in the literature, but its potential has not been appreciated by modern genomic researchers. Thus, the chief aim of therapeutic MBTs is believed to ensure that *tridosha* remains in homeostatic state [22]. Some studies have found that chanting of *maha mantra* results in perfect harmony among the *gunas* besides reducing the stress and depression [23].

### 2.2. Physiological Aspects of MBTs

Current scientific evidence suggests MBTs as a potential strategy in enhancing immunity because the benefit of MBTs on various body systems comprising of nervous, cardiorespiratory, musculoskeletal, reproductive, and endocrine systems, etc. has been shown to alter stress, depression, and anxiety [14,24]. Studies indicate that the prolonged stress conditions lead to the continuous release of stress hormones disrupting the hypothalamus-pituitary-adrenalin axis. It is argued that this system can be maintained by activating the regulation of parasympathetic nervous system through the release of endorphins induced by MBTs [25]. Even short sessions of various MBTs can bring substantial changes in neurotransmitters of brain but whether it has direct or indirect correlation to *guna*, *prakriti,* or the clinical personality types, described in medical literature, has not been examined. Yoga intervention has shown significant improvement in various physiological and psychological health issues among soldiers posted at high altitudes when compared with the control group that were engaged in carrying out routine activities for physical training. [26] Coupled with evidence that the breathing processes can modulate cardiac autonomic tone and improve psychological status, there is possibility that this parasympathetic activity maintains a sympatho-vagal balance among SKY or yoga practitioners [27]. This can be analyzed as a large number of individuals practice SKY. This is important because yoga training in subjects above 40 years of age has helped in maintaining age-related cardiovascular functions by significantly reducing the resting pulse rate, systolic and diastolic blood pressure and increasing the valsalva ratio further leading to an increase in baroreflex sensitivity [28]. Furthermore, a significant increase in reduced glutathione/oxidized glutathione ratio and total antioxidant status (TAS) has confirmed the beneficial effect of yoga training on the antioxidant system of the body [29].

Various other MBTs like meditation, yogic breathing exercise, Chinese form of meditation, qigong etc. helps in providing relief by reducing stress and timely elimination of free radicals from the body. It is argued that necrosis, stenosis, tissue starvation and conditions of hypoxia [30] can also be prevented. For this reason, traditional Chinese medicine (TCM) has been integrated with modern therapies in Chinese hospitals. Kozo nishino, a breathing method developed in 1970s, has been similarly found effective in reducing the stress levels and in improving immune-regulatory systems of practitioners [31]. Studies have shown that yogic breathing meditation can directly optimize human performance by improving metabolism, immune system and central nervous system functioning. A breath-based meditation sequence such as SKY also helps in regulation of vascular tension and respiration rate in subjects besides stimulating self-awareness, human performance, and happiness. The mechanisms by which yogic breathing positively impacts the individual’s compensatory reserves are not yet completely understood [32] because molecular and ayurgenomic approaches in combination with in vitro and in vivo studies have so far not been examined. The effect of yoga and meditation-based lifestyle intervention on cellular aging in apparently healthy individuals has also been elucidated in considerable detail. A significant improvement in biomarkers of cellular aging including DNA damage markers, oxidative stress markers and total antioxidant capacity (TAC) has been reported. Thus, by adopting MBTs intervention, the reversal of various markers responsible for the aging process has been observed [33], generating hope for the future of cost-effective MBTs. Furthermore, practicing falun qigong, a Chinese form of MBT helps in increasing immunity and metabolic rate in addition to decreasing cell death of the regulatory genes [34].

A specific *Pranayama*, called alternate nose breathing (ANB), was examined by the deep breathing test, orthostatic tolerance test and heart rate (HR interval) revealing that it may exert its effects on the parasympathetic nervous system of young healthy participants [35]. In addition, the s*havasan* (corpse posture) and s*avitri Pranayama* (slow and deep breathing in supine position) has been shown to decrease the oxygen consumption, heart rate and diastolic blood pressure due to the reduction in the premature ventricular complexes [14]. It is also hypothesized that *Pranayama* positively impacts the management of coronary artery disease patients [36]. Unfortunately, none of these studies have examined the molecular and epigenetic changes underlying this correlation.

Although several studies have demonstrated the beneficial effects of various MBTs in maintaining physiological and psychological health, extensive research is required to test the preventive effect of yoga and meditative practices at high altitudes through molecular, longitudinal and comparative studies [37].

Various studies including randomized controlled trials (RCTs) have examined the impact of MBTs on an individual’s health parameters [38,39,40,41,42,43,44]. Table 1 summarizes the important studies depicting the positive impact of MBTs. However, these studies ignore the importance of *triguna* or *tridosha* parameters.

Furthermore, other scientific studies as described in Table 2, have examined the intervention of MBTs especially in hypoxic conditions on human health albeit without integration of *Prakriti* analysis [26,45,46,47].

Even though various MBT-based scientific studies have been linked to cognition, imaging, biochemical profiling and physiological changes yet the investigations at molecular or ayurgenomic level are lacking.

The following section describes the molecular findings studied so far in the context of MBTs.

### 2.3. Molecular Basis for MBTs

Available evidence proves that increased telomerase activity and stem cells count in peripheral blood may increase longevity and better quality of life at later age as shown in an intensive mind and body therapeutic program among retreat participants [48]. Similarly, another study described the effect of meditation on telomerase activity in retreat participants when compared to controls [49]. However, it is still not clear whether the increased regenerative capacity, claimed in this study, is a result of exercise induced by pulmonary activation of stem cells or otherwise. Animal-based mechanistic studies in this field are also lacking. It has also been shown that there is hypomethylation of the TNF-*α*, as compared to other inflammatory markers like IL-6, in a yoga group [50]. However, its analysis in the context of *prakriti* or *guna* principles and/or physiological parameters has never been undertaken. Available evidence indicates that the hypoxia induced by high altitude alters homeostasis and the quality of sleep. It has even been shown that a key oxygen sensor, EGLN1, varies from person to person due to unique *prakriti* constitution which has led to the discovery of therapeutic interventions by use of ayurgenomics approach in future hitherto unexplored [17,18]. These alterations might either be the result of stimulation of erythropoietin or inflammatory markers like IL-1, IL-6, and TNF-*α* [51] which may themselves get altered. This provides an attractive research question for understanding the effect of MBTs in the context of Ayurgenomic principles. For example, because it has been shown that the decrease in plasma VEGF, at high altitude, is associated with acute mountain sickness (AMS) or hypoxia. It was subsequently hypothesized that the subjects who do not develop AMS might have higher level of plasma VEGF levels at high altitudes. This may even have opposite effects at sea-level [52]. It has also been demonstrated that with the intervention of MBTs, the expression of BDNF increases. The key role of BDNF is to maintain brain neuroplasticity, stress, rescue depression and certain other brain disorders [53,54]. Therefore, an altered hypoxia-BDNF interaction could be an important cause underlying diseases like pulmonary hypertension [55] at high altitudes. Even the in vitro and in vivo experiments of intervention of MBTs are lacking. The impact of various MBTs (such as SKY) has shown differential gene expression in aging, apoptosis, stress-related signaling and metabolic pathways. This suggests the generality of need to understand the cellular and molecular basis of cell longevity in SKY practitioners at high altitudes [56]. Existing evidence also points out that micro-RNAs play an important role in development and functioning of brain and have recently emerged as neuroprotective agent under hypoxic/ischemic conditions [57,58]. Cozzolino et al. reported a heterozygous to homozygous shift of epigenetic profile in individuals experiencing mind-body therapy, though the high altitude component was missing [59]. Moreover, the effect of hypoxia induced hypermethylation have been associated to cardiac cell fibrosis suggesting its role in the high altitude [60]. A comprehensive study that examines whether MBTs can bring epigenetic changes at high altitude and whether these studies can predict vulnerability to AMS based on molecular markers remains unexplored. Once these studies are executed, the effect of MBTs can be tested. Besides, most related studies have either very small sample size or carried out without inclusion of related physiological, *prakriti* and/or clinical parameters.

### 2.4. Efficacy of MBTs in Neuroprotection

Yogic practice plays an important role in the central nervous system. Studies underlying the neuroprotective effects of long-term yoga have been reported. It has been shown that gray matter volume increases significantly among experienced yoga practitioners as compared to controls [61]. Moreover, the impact of duration (yearly and weekly) and frequency of practicing yoga and its different aspects also influences specific brain regions [62]. Yoga performed for a short and long period enhances the volume of gray matter in brain. These regions are involved in cognitive and motor functions [63,64,65,66,67,68,69]. It has been reported that different aspects of yoga, including meditation, postures and breathing techniques act differently on brain. These are associated with alterations in various parts of the brain. This is indicated by an increase in gray matter and hippocampal volume [70,71,72,73], which is known for memory and learning, insular [66,70,74] and left inferior temporal gyrus [70,71,75] volume as compared to controls. These findings are fascinating and reveal the powerful effects of meditation in altering neuroplasticity even though it is known that after a certain period brain structure stops changing. Furthermore, the gray matter density in the amygdala, a region linked with anxiety and stress, tends to shrink after mindfulness meditation. Similar results have been obtained in a study demonstrating greater hippocampal and prefrontal gray matter density upon physical activity [76]. However, these studies need to be carried out with the help of molecular and ayurgenomic tools in order to understand these mechanisms. As aging is another factor which reduces gray matter density of the whole brain, [77,78,79] the effect of mindfulness meditation and other yoga-related techniques on age-related reduction of gray matter has also been shown in detail [80,81,82].

Modern day stress or stress generated due to hypoxic environment at high altitude are both related to the structural and functional alterations in brain regions, resulting into various psychosocial problems including reduction in cognitive abilities. Several studies have reported cognitive enhancement due to various *pranayama* [83].

A study used a cognitive screening test on adapted lowlanders staying at high altitudes since more than one year, and it showed the increased prevalence rate of mild cognitive impairment (MCI), decline in working memory and imbalance in mind-body co-ordination [84]. However, due to prolonged deprivation of oxygen (hypoxia/ischemia) in the brain, many diseases like stroke, Alzheimer’s, encephalopathy etc., may onset. It is important to identify the alternate therapeutic strategies to alleviate the pathophysiological conditions resulting from high altitude-related conditions [15,85,86]. A recent clinical trial tested the beneficial impact of meditation in Amyotrophic lateral sclerosis (ALS) population and it was observed that there was a significant improvement in their quality of life and psychological health [87]. Further investigations have shown the enhanced verbal task and spatial task performance as a result of unilateral nostril breathing. These findings indicate that the yogic breathing may have a potential effect in treating psycho-physiological disorders [88,89]. SKY has been shown to be a good alternative of medication for stress management as it helps in the improvement of human stress tolerance and enhancement of cognitive performance [90]. In addition, it has been found that the intervention of MBTs leads to increase in the cognitive functions, with concomitant increase in GABA levels further leading to the reduction of anxiety of the practitioners [91,92].

Even though various studies on yoga and meditation have been shown to exert health-promoting benefits yet these have not been studied for acclimatization at high altitude.

### 2.5. Efficacy of MBTs at Extreme Environments

Evidence demonstrates that vigorous exercise (10 h a day) in Antarctica by two men for 95 days, with energy expenditure of 21.3 MJ per day, showed a decline in maximal O_2_ consumption, isometric force production in different muscles, cytoplasmic and skeletal muscles’ enzymes activities. Although analysis of their plasma samples showed normal cholesterol and triglycerides, decrease in glucose, increase in insulin levels, and decrease in testosterone and luteinizing hormone were noticed [93]. In addition, another study in Antarctica on 7 subjects analyzed how weight loss exceeded unexpectedly as the energy requirements increased [94]. Furthermore, a study conducted on 9 Mt. Everest climbers after 7 weeks of climbing at high altitude showed reduced bodyweight, increased activity of growth hormone (GH)/insulin-like growth factor-I (IGF-I) axis. The expected increase in ghrelin and leptin levels was not observed. Hence, it was assumed that weight loss could be the chief cause for it [95]. The role of yoga on 15 experienced females has shown that it enhances thermal stress but is well tolerated by this group of female [96].

### 2.6. Drugs or MBTs for High Altitude?

Drugs like acetazolamide, dexamethasone, nifedipine are recommended for early acclimatization. Symptomatic therapies include aspirin, ibuprofen for headache and Promethazine for nausea. The mechanism of action of this drug shows that it may affect kidney, RBCs, lungs and brain and may even be contraindicated in the existing pathophysiology of individuals [97,98]. Studies designed to examine the role of pre-conditioning by MBTs for early acclimatization can only be undertaken if there is political will to launch Integrative Medicine in premier Medical Institutes.

Long term studies that examine MBTs in normoxic as well as hypoxic conditions, are required by both in vitro and in vivo analysis.

### 2.7. In Vitro Hypoxia Studies

High altitudes are characterised by significant drop in the atmospheric pressure, which results in hypobaric conditions. The hypobaric conditions are accompanied by decrease in the oxygen percentage at considerable heights above sea-level and this may cause ischemia. The ischemic cascade is usually marked with the reduction in blood flow and drop in the energy supply to the tissue level [99], which results in the oxidative stress mediated by free radicals, excitotoxicity and apoptosis [100,101]. To induce hypobaric ischemia into the cells in vitro, the cultures are exposed to hypoxia [102], glucose deprivation [103] and/or employing both the methods together to achieve a combinatorial response i.e., oxygen and glucose deprivation (OGD) [104]. Excitotoxic conditions are mimicked by the overexpression/injection of glutamate/N-methyl-D-aspartate (NMDA) [105]. Sodium nitroprusside and hydrogen peroxide are essential compounds for inducing oxidative stress [106,107]. Studies suggest that the exogenous administration of tumor necrosis factor-α (TNF-α) results in cell damage mediated by nuclear factor kappa (NF-κB) [108].

Hypoxia or hypobaric hypoxia can incite a complex trigger in ischemia at various organ/tissue. The affected organs include cardiovascular and nervous systems. Both the systems are high on oxygen and glucose with high metabolism and energy consumption. Cardiomyocytes exposed to prolonged periods of hypoxia are induced to hypertrophy. Such hypertrophy leads to impaired mitochondrial homeostasis causing pathological damage [109,110,111]. The nuclear genome in general encodes proteins for the regulation of mitochondrial functions such as electron transport chain (complexes I–V) [112]. The hypoxic stress induces malfunctioning which causes cell damage and promotes excessive leakage of free radicals (complexes I and III) [113], imbalance in the energy metabolism of the cell (complexes II and IV) [114] and deficiency in the bio-energy reserve of the cell (complex V) [115]. The association of impaired electron transport chain complexes to cardiovascular and neurovascular damage [116] have not been examined post MBTs, in extreme climates.

Hypoxic ischemia in the CNS/brain leads to fatal complications and co-morbidities viz. epilepsy, palsy and cognitive decline [117,118,119]. Excessive L-glutamate has been reported to cause apoptosis in the CNS γ-aminobutyric acid (GABA) is known to counter the activity of L-glutamate [120]. Hypoxia tends to increase the VEGF levels in the systemic circulation and causes angiogenesis, which is pathological and leads to diseases like age-related macular degeneration (AMD) and cancer. Supplementing cell-cultures with agents/factors antagonising the pro-inflammatory proteins have been studied but not with sera derived pre and post MBT. Melatonin is known to be secreted and postulated to improve the cardiorespiratory and psychological profiles in individuals practicing hatha yoga and meditation [121]. In this case, when the cells in vitro were supplemented with melatonin, it was shown to regulate the angiogenic and inflammatory proteins [122]. These studies should re-focus our attention towards the importance of studying the effects of anti-inflammatory factors on these pathologies in vitro. Serum derived from individuals practicing yoga/meditation can be used to supplement/condition the growth media of in vitro pathology models so that the effects can be mechanistically analyzed for developing preventive or curative solutions.

### 2.8. Animal Models of Exercise and Meditation

Existing animal model studies support the amelioration of hypoxia-related effects through exercise and physical activity. Ding et al. demonstrated that exercise preconditioning (30 min of exercise on treadmill daily for 3 weeks) in stroke induced by middle cerebral artery occlusion model (MCAO) in adult male Sprague Dawley rats was beneficial. It was noticed that in rats that were exposed to exercise, the size of the infarct was diminished and the expression of TNF-α receptors was also milder as compared to control rats [123]. Cheng et al. examined the aged mice that underwent surgery subsequent to swimming (for one hour daily), angiogenesis to hypoxia was evident [124]. Similarly, Zucker rats (lean and obese male rats) were analyzed for the effects of exercise on abdominal fat and the expression of vascular endothelial growth factor A (VEGF-A) and hypoxic marker (lactate) was estimated. Lean and obese rats were selected and allocated to sedentary and exercise groups randomly. It was found that exercise (5 times per week walk on treadmill for 8 weeks) raised VEGF-A in adipose tissue, whereas it decreased lactate in adipose tissue of obese rats [125]. Like exercise, meditation in the form of lower-frequency oscillation rhythms has also been shown to have positive effects on the mouse anterior cingulate cortex (ACC). It was found that rhythmic stimulation of brain reduced anxious behavior in them [126]. Studies on other animals like sheep and goats have also shown the positive effect of exercise in countering hypoxia as it helps in increasing the cardiac output and lung lymph flow at normobaric as well as hypobaric conditions. This is believed to be the result of increasing the vascular surface area of lungs [127]. There is existing evidence that indicates a key role of voluntary exercise in inducing hippocampal neurogenesis, telomerase activity, cellular effects and behavioral effects in rodent models of schizophrenia, however, comprehensive molecular studies are required for a deeper understanding of whether MBTs may directly influence neurogenesis in humans and if these can act as potential therapeutic interventions for the treatment of high altitude sickness, Diabetes or Schizophrenia [128]. The animal models could be used in future research to test the efficacy of MBTs in cell survival mechanisms.

## 3. Conclusions

The detrimental effects of hypoxia that result from the production of free radicals in extreme climates may cause pulmonary or cerebral edema, affect skin-fold thickness, and alter limb circumference besides impacting alertness, memory, and reasoning. It must be emphasized that molecular changes precede such physiological or clinical changes, regardless of the organ involved and provide early information for interventional targets. Even the change in diastolic blood pressure, heart rate and increase in high-density lipoprotein (HDL) cholesterol occur by preceding cellular changes that can be detected by sophisticated molecular and genetic tools, one such tool which can be used to detect these changes is ayurgenomics which, for example, can be used to assess VEGF and BDNF alterations in various MBT practitioners. Modeling of such conditions in vitro or in vivo may help in developing mechanistic insights of MBT intervention, either through short term studies or RCTs. A deeper insight into the molecular mechanisms and epigenetic changes underlying the effects of consistent use of MBTs will help to apply these MBTs not only for general wellness of the public in disease prevention but also as a targeted therapeutic measure that may be used alone or in conjunction with other allopathic modalities of treatment. A deeper understanding is required not just on the underlying mechanisms but also in developing a standardized protocol that may be used in patients with these pathological states. This will not only be useful to mountaineers, miners, submariners, deep sea divers, sky divers, and astronauts, but also for the general health of individuals.

## Figures and Tables

**Table 1:** Summary of studies examining effect of MBTs on body.

Study	Sample Size & Treatment Groups	Intervention	Targeted Output	Outcome Measurements	Findings
Cramer et al. 2014	*n* = 3168 from 44 RCTs	Meta-analysis of yoga RCT	Cardiac function, lipid profiles	Diastolic and systolic blood pressure, heart rate, respiratory rate, waist and hip circumference, waist/hip ratio, lipid profile and HbA1c	Clinically important effect of yoga on cardiovascular diseases and associated risk factors
Innes and Vincent 2007	15 uncontrolled trials, 6 NRCTs and 4 RCTs	Systematic review on yoga	Type 2 Diabetes mellitus (T2DM)	Glucose tolerance, insulin sensitivity, lipid profiles, anthropometric characteristics, blood pressure, oxidative stress, coagulation profiles, sympathetic activation, and pulmonary function	Yoga is Beneficial for improving indices of risk in adults with T2DM and in preventing and managing cardiovascular complications
Newberg et al. 2010	14 subjects with memory problems	8-week meditation program	Memory and Cerebral blood flow (CBF)	SPECT scan andNeuropsychological assessment of verbal fluency, trails B and logical memory	Meditation leads to significant increase in baseline CBF ratios in the prefrontal, superior frontal and superior parietal cortices
Froeliger et al. 2012	*n* = 14; 7 Hatha yoga meditation practitioners and 7 matched control group	Hatha yoga meditation	Voxel-based morphometry (VBM), Cognitive Function, Anxiety, Stress and anger	Cognitive failures questionnaire (CFQ), Beck Anxiety Inventory, 20-item positive and negative affect schedule (PANAS)	Positive correlation of greater gray matter volume with hatha yoga meditation and significant improvement in cognitive failures task
Hernández et al. 2016	*n* = 23 Sahaja yoga Meditators; *n* = 23 age, sex education matched non-meditators	Long-Term Sahaja yoga meditation	Grey matter volume	Structural magnetic resonance imaging	Sahaja yoga leads to overall larger grey matter volume and regional enlargement in several right hemispheric cortical and subcortical brain regions
Neuendorf et al. 2015	13/23 studies using meditation, 21/30 using movement MBIs, and 14/25 using relaxation	Review of mind-body interventions (MBIs) on sleep	Sleep outcome measure	Subjective and objective sleep outcomes, sleep quality, sleep duration, and sleep latency.	MBIs as a treatment for sleep disorders
Pomidori et al. 2009	*n* = 11, chronic obstructive pulmonary disease (COPD) patients.	Yoga with deep and slow breathing	Ventilatory pattern and oxygen saturation (SaO2%	SaO2% and respiratory parameters including tidal volume, VE, respiratory rate, inspiratory time, total breath time and fractional inspiratory time	Yoga leads to significant improvement in SaO2% and induction of favorable respiratory changes in COPD patients.

**Table 2:** Studies showing the impact of MBTs.

Study	Sample Size & Treatment Groups	Interventions	Targeted Output	Outcome Measurements	Findings
Bernardi et al. 2001	*n* = 19; 10 yoga trainees and 9 controls	Slow yogic Breathing	Blood oxidation and sympathetic system	minute ventilation (VE), oxygen saturation, RR interval, blood pressure and response to carotid baroreceptors at baseline and after acute hypobaric hypoxia	Slow yogic breathing maintains better blood oxygenation and reduces sympathetic activation in hypoxic conditions
Bernardi et al. 2007	*n* = 75; 12 Caucasian yoga trainees, 12 control sea-level residents, 38 active lifestyle high altitude natives and 13 high altitude residents performing yoga like respiratory exercises	Yoga	Ventilatory, cardiovascular and hematological parameters	hypoxic ventilatory response (HVR), red blood cell count, hematocrit, blood pressure, RR interval, minute ventilation	Yoga pre-conditioning leads to few hematological changes, maintains oxygen and induces respiratory adaptations to cope up with altitude-induced hypoxia in sea-level residents
Bilo et al. 2012	*n* = 67; 39 at 4559 m and 28 at 5440 m	Slow deep breathing	Oxygen saturation, pulmonary and systemic hemodynamics	Minute ventilation or pulmonary CO diffusion, Spo2, systematic, and pulmonary arterial pressure	Slow deep breathing increases SpO2, which improves ventilation efficiency and reduces systematic and pulmonary arterial pressure at high altitude
Himashree et al. 2016	*n* = 200 Indian army soldiers; 100 performed physical training and 100 Yoga at 3445 m	Yoga	Physiological and biochemical status	Height and Weight, body fat percentage, heart rate, respiratory rate, systolic and diastolic blood pressure, peripheral saturation of oxygen, end tidal CO2, pulmonary functions, hematological variables lipid profile serum urea, creatinine, liver enzymes, blood glucose, and anxiety scores.	Significant improvement in health indices and performance was observed in yoga group as compared to control group at high altitude
